# Evaluation of the Probiotic Strain *Bifidobacterium longum* subsp. *Infantis* CECT 7210 Capacities to Improve Health Status and Fight Digestive Pathogens in a Piglet Model

**DOI:** 10.3389/fmicb.2017.00533

**Published:** 2017-04-11

**Authors:** Emili Barba-Vidal, Lorena Castillejos, Paola López-Colom, Montserrat Rivero Urgell, José A. Moreno Muñoz, Susana M. Martín-Orúe

**Affiliations:** ^1^Animal Nutrition and Welfare Service, Departament de Ciència Animal i dels Aliments, Universitat Autònoma de BarcelonaBellaterra, Spain; ^2^Laboratorios Ordesa S. L., Investigación BásicaBarcelona, Spain

**Keywords:** probiotic, pig-model, *Salmonella* Typhimurium, *Escherichia coli*, microbiota, diarrhea, infant

## Abstract

Probiotics have been demonstrated to be useful to enhance gut health and prevent gastrointestinal infections. The objective of this study is to demonstrate the potential of the probiotic strain *Bifidobacterium longum* subsp. *infantis* CECT 7210 (*B. infantis* IM1) to prevent and fight intestinal disease by using a *Salmonella* Typhimurium (Trial 1) or an enterotoxigenic *Escherichia coli* K88 (Trial 2) oral challenge in a weaning piglet model. Seventy-two piglets were used in each trial. After an adaptation period, animals were orally challenged. One animal per pen was euthanized at Days 4 and 8/9 (Trial 1/Trial 2) post-inoculation (PI). Animal performance, clinical signs, pathogen excretion, fermentation, immune response, and intestinal morphology were evaluated. In Trial 1, most parameters responded to the challenge, whereas, in Trial 2, effects were much milder. Consistent effects of the probiotic were detected in both experiments: Reduction of pathogen excretion (*P* = 0.043 on Day 3 PI, Trial 1) or ileal colonization (33% reduction of animals with countable coliforms; *P* = 0.077, Trial 2); increases in intraepithelial lymphocytes (*P* = 0.002 on Day 8 PI in Trial 1, *P* = 0.091 on Day 4 PI in Trial 2), and improvement of the fermentation profile by increasing butyric acid in non-challenged animals [*P* challenge × probiotic (interaction) = 0.092 in Trial 1 and *P* = 0.056 in Trial 2] concomitant with an enhancement of the villus:crypt ratio on Day 8/9 PI (*P* interaction = 0.091 for Trial 1 and *P* = 0.006 for Trial 2). Challenged animals treated with the probiotic showed reduced feed intakes (*P* interaction = 0.019 in Trial 1 and *P* = 0.020 in Trial 2) and had lower short-chain fatty acid concentrations in the colon (*P* interaction = 0.008 in Trial 1 and *P* = 0.082 in Trial 2). In conclusion, this probiotic demonstrated potential to reduce the intestinal colonization by pathogens and to stimulate local immune response. However, effects on feed intake, microbial fermentation, and intestinal architecture showed a differential pattern between challenged and non-challenged animals. Effects of the probiotic intervention were dependent on the structure of the ecosystem in which it was applied.

## Introduction

Gastroenteritis due to enteric infections occur globally each year, and enterotoxigenic *Escherichia coli* spp. (ETEC) and *Salmonella* spp. are among the most common bacterial causes of diarrhea-associated morbidity and mortality (CDC, 2013; Lanata et al., 2013), especially in children up to 5 years of age (Payment, 2002; Liu et al., 2012; Kotloff et al., 2013). The estimated annual mortality from illness due to *Salmonella* spp., considering the global population, rises to 155,000 deaths (Majowicz et al., 2010), and 157,000 deaths are annually associated with ETEC only in children from 28 days to 5 years of age (Bourgeois et al., 2016); this represents, on the whole, a considerable burden in both developing and developed countries.

It has largely been demonstrated that breast-feeding prevents gastrointestinal diseases in infants and can also confer protection from translocation of intestinal pathogens across the gut mucosa (Wold and Adlerberth, 2000). However, lifestyle in developed countries is leading to a drastic decrease in breast-feeding and, although milk formulae have been very much improved during the last few decades, they are still far from emulating the multitude of biological functions of mother's milk.

There are well-documented benefits of administering probiotic microorganisms in milk formulas which include improvements in several infections, allergic disorders, diarrhea, and inflammatory diseases (Bin-Nun et al., 2005; Minocha, 2009). Furthermore, probiotics and their metabolites have been suggested to have an important role in the formation or establishment of a well-balanced, indigenous intestinal microbiota in new-born infants, and adults (Gill, 2003; Salazar et al., 2009) and to be remarkably beneficial in improving microbiota in hospitalized pre-term infants (Schwiertz et al., 2003).

In relation to the efficacy of probiotics to fight infectious diseases, various probiotic strains, mainly from the *Lactobacillus* spp. and *Bifidobacterium* spp. genus have demonstrated their potential to inhibit or ameliorate the outcome of these infections in humans and animal-models (Gill et al., 2001; Weizman et al., 2005; Spinler et al., 2008; Tanner et al., 2016). In particular, *Bifidobacterium longum* subsp. infantis CECT 7210, brand name *B. infantis* IM1, which was isolated from infant feces, has previously demonstrated a protective effect against rotavirus infection *in vitro* and in a murine model (Moreno Muñoz et al., 2011).

Pig *in vivo* models have usually been selected as an excellent animal model to study various microbial infectious diseases due to its greatest similarity to humans in terms of anatomy, genetics, and physiology (Meurens et al., 2012). Moreover, the domestic pig is susceptible, like humans, to ETEC and non-typhoidal *Salmonella enteritidis* serovars such as Typhimurium and Enteritidis, as these pathogens have broad host specificity (Blanco et al., 1991; Gal-Mor et al., 2014).

The objective of this work is, therefore, to demonstrate the potential of a probiotic strain, *B. longum* subsp. *infantis* CECT 7210, to enhance gut health at early-life stages and to fight diarrhea-related diseases caused by ETEC K88 or *Salmonella* by using the weaning piglet as a model.

## Materials and methods

Two different experiments were performed to evaluate efficacy of the probiotic against an oral challenge with *Samonella* Typhimurium (Trial 1) or ETEC K88 (Trial 2). Both trials were performed at the Experimental Unit of the Universitat Autònoma de Barcelona (UAB) and received prior approval (permit no. CEAAH1619) from the Animal and Human Experimental Ethical Committee of this institution. The treatment, management, housing, husbandry and slaughtering conditions conformed to the European Union Guideline (Directive 2010/63/EU)^1^.

### Animals, housing, and experimental design

The two trials were conducted as a Level 2 High-Risk Biosecurity Procedure, with appropriate training of the personnel involved. A total of 144 male piglets were used: 72 Large White × Landrace piglets weaned at 24 (±4) days of age and 7.9 (±0.05) kg body weight (BW) for the first trial and 72 Landrace piglets weaned at 21 (±2) days of age and 6.8 (±0.19) kg BW for the second. In both cases, animals were obtained from high-sanitary-status farms, in the first trial from mothers serologically negative to *Salmonella*, and, in the second trial, from mothers that did not receive any *E. coli* vaccination.

The UAB facilities available for these studies were an experimental unit with three rooms of eight pens each (twenty-four pens, three animals per pen). In each trial, animals were distributed by taking initial BW into account for a similar average BW within pens. The pens were allocated to four treatment groups following an unbalanced 2 × 2 factorial arrangement (factors being probiotic and pathogen challenge), with eight replicates per treatment for the challenged animals and four replicates for the non-challenged group. The treatments were, therefore: (1) no challenge + no probiotic (NN); (2) no challenge + probiotic (NP); (3) challenged + no probiotic (CN); and (4) challenged + probiotic (CP). Two rooms were challenged with pathogens and one was left unchallenged. In each room, probiotic treatment was distributed within four pens on one side of the room, and the four control pens were on the other side of the room, separated by a corridor in between.

Pigs were maintained under a 14:30 h light/ 9:30 h dark lighting regimen. Each pen (2 m^2^) had a feeder and a water nipple to provide feed and water for *ad libitum* consumption. The weaning rooms were equipped with automatic heating, forced ventilation, and an individual heat-light per pen. The trials were conducted during the spring season (March for the first trial and May for the second), with an average room temperature of 26°C (± 4°C).

### Probiotic strain and diets

The probiotic treatment was supplied by Ordesa S.L. and consisted of a daily dosage (10^9^ colony-forming units [cfu]) of *B. longum* subsp. infantis CECT 7210, which was supplemented in a 2 mL solution, while the control group received a solution of the same amount of carrier as placebo. During the experimental period, pigs received the treatment orally and individually, in a daily pattern using disposable syringes without needle. The probiotic tested was a unique batch of lyophilized bacteria, which was re-suspended every day no more than 1 h prior to administration. A pre-starter diet without additives (Supplementary Table ST.1) was formulated to satisfy the nutrient requirement standards for pigs (NRC, 2012) and was given in a mash form.

### Salmonella and ETEC strains

In the first trial, the bacterial strain used for the oral challenge was a *Salmonella* Typhimurium var. Monophasic (formula: 4,5,12:i:-, resistance profile: ACSSuT-Ge, Fagotype: U302) isolated from a salmonellosis outbreak of fattening pigs in Spain (mainly enteric and with sporadic septicemia), which was provided by the *Infectious Diseases Laboratory (Ref. 301/99)* of the UAB. The oral inoculum was prepared by 24 h incubation at 37°C in buffered peptone water (Oxoid; Hampshire, UK) and diluted (1:20) with sterile phosphate buffered saline (PBS) (Sigma-Aldrich; Madrid, Spain). Final concentrations of the inocula were 1 × 10^9^ cfu/mL for the first inoculation day and 3 × 10^9^ cfu/mL for the second day. Inocula concentrations were determined before the inoculation by McFarland standards and were plated the same day in order to check them by manual plate counting.

In the second trial, the bacterial strain of ETEC K88 used (serotype O149:K91:H10 [K-88]/LT-I/STb) was isolated from a colibacillosis outbreak in Spain (Blanco et al., 1997). It was kindly donated by the Dr. Blanco *E. coli* Reference Laboratory, Veterinary Faculty of Santiago de Compostela, Lugo (Reference FV12048). The oral inocula were prepared by an overnight incubation at 37°C in Brain Heart Infusion broth (Oxoid; Hampshire, England) with slow agitation (1 × g) in an orbital incubator. For the first inoculation, the culture was given directly, and for the second inoculation day, bacteria were concentrated by centrifuging (2,000 × g, 10 min and 4°C), and supernatant was eliminated. Final concentration of the inocula was 9 × 10^8^ cfu/mL for the first inoculation day and 8 × 10^9^ cfu/mL for the second day. Inocula concentrations were also determined before the inoculation by McFarland standards and were plated the same day for manual plate counting.

### Experimental procedure

The duration of the study was 16 days for the *Salmonella* Trial and 14 days for the ETEC K88 Trial. After an adaptation period of 7 days for Trial 1 or 4 days for Trial 2, the animals were orally challenged with the pathogen. One animal of each pen was euthanized on Days 4 and 8 post-inoculation (PI) in Trial 1 and on Days 4 and 9 PI in Trial 2.

Fecal samples for microbiological analysis were obtained from random animals at their arrival, and individual body weight and pen feed consumption were registered during the adaptation time. After the adaptation period, the pathogenic bacteria culture was administered to the challenged group by oral gavage: two 2 mL doses (2 × 10^9^ and 6 × 10^9^ cfu) of *Salmonella* Typhimurium on Days 8 and 10 in the first trial and two 6 mL doses (5 × 10^9^ and 5 × 10^10^ cfu) of ETEC K88 on Days 5 and 6 in the second trial. The same amount of sterile broth was given to the non-challenged animals. In order to ensure that the stomach was full at the time of inoculation, and thus, facilitate bacterial colonization. Feed withdrawal was performed at 21:00 h of the previous day and provided again 15 min before inoculation.

From the first challenge onwards, animals were checked daily for clinical signs to evaluate their status post-inoculation (i.e., dehydration, apathy, and fecal score), always by the same person. Fecal score was measured using a scale: 1 = solid and cloddy, 2 = soft with shape, 3 = very soft or viscous liquid and 4 = watery or with blood. Rectal temperature was assessed with a digital thermometer (Thermoval Rapid, Hartmann; Spain) on Days 1, 2, and 3 PI. Mortality rate was also registered, and no antibiotic treatment was administered to any of the animals in the trials.

Body weight was recorded on Days 0, 4, and 8 PI (9 PI in the case of ETEC K88), while feed consumption on Days 0, 1, 2, 4, 6, and 8 PI (9 PI in the case of ETEC K88). The average daily gain (ADG), average daily feed intake (ADFI), and gain:feed ratio (G:F) were calculated by pen.

For microbiological analysis, on the inoculation day (0 PI) fecal samples were taken aseptically from 24 animals after spontaneous defecation associated with the manipulation of the animal or by digital stimulation. In the case of the *Salmonella* trial, fecal samples were taken from the animal with the highest initial BW of each pen (*N* = 24), while in the ETEC K88 challenge, fecal samples were obtained from the animal with the medium body weight of each pen (*N* = 24). For the *Salmonella* trial, additional fecal samples were collected on Days 1, 3, and 7 PI from the same animal.

On Days 4 and 8 PI (9 PI in the ETEC K88 challenge), one pig per pen was euthanized. On Day 4 PI, the animal selected was the one with the intermediate initial BW, while on Day 8/9 PI, the heaviest was selected. Animals were euthanized and sequentially sampled during the morning (between 08:00 and 12:00 h). Prior to euthanasia, a 10 mL sample of blood was obtained by venipuncture of the cranial vena cava using 10 mL tubes without anticoagulant (Aquisel; Madrid, Spain). Immediately after blood sampling, piglets received an intravenous, lethal injection of sodium pentobarbital (200 mg/kg BW; Dolethal, Vetoquinol S.A.; Madrid, Spain). Once dead, the animals were bled, the abdomen was immediately opened and the whole gastrointestinal tract excised.

In the ETEC K88 trial, fecal samples were obtained directly from the rectum for traditional microbiology. For both trials, digesta (~50 mL) from the ileum and proximal colon (considered to be 0.75 m from the ileocecal junction) was collected and homogenized. The pH of the contents was immediately determined with a pH-meter calibrated on each day of use (Crison 52–32 electrode, Net Interlab; Barcelona, Spain).

From colonic digesta various subsamples were taken for different analysis. One aliquot was stored at −80°C for ETEC K88 quantification by qPCR. To determine the presence of the probiotic in the gut, additional subsamples were taken. Bacterial isolation was performed in these samples before storing at −80°C with *Geniul* commercial protocol (Terrassa, Spain). Briefly, 1 g of colonic content sample was weighed in a 15 mL Falcon tube and diluted 1:10 with enriched Man Rogosa Sharpe (MRS) broth (Oxoid; Madrid, Spain) + 0.25% cysteine (Sigma-Aldrich; Madrid, Spain) + 2% Tween 80 (Sigma-Aldrich; Madrid, Spain). Ten glass spheres (5 mm diameter) were added to the tube and vortex (1 min) to homogenize the suspension. Two-hundred-fifty microliters of the sample suspension were transferred to an Eppendorf tube with 250 μL of enriched MRS broth. Three centrifugation (13,000 × g for 5 min at 4°C) and re-suspension (500 μL of enriched MRS broth) steps were performed and, finally, the bacterial pellet was re-suspended in 200 μL of sterile PBS and stored at −80°C for DNA extraction.

A set of ileal and colonic digesta samples was also preserved in a H_2_SO_4_ solution (3 mL of content plus 3 mL of 0.2 N H_2_SO_4_) for ammonia (NH_3_) determination and was kept frozen at −20°C. An additional ileal and colonic sample set (~20 g) was also frozen (−20°C) until analyzed for short-chain fatty acids (SCFA) and lactic acid.

Bacteria attached to the intestinal mucosa were also analyzed in the ETEC K88 trial. For that, 5-cm-long sections of distal ileum were collected from each animal, washed thoroughly with sterile PBS, opened longitudinally and scraped with a microscopy glass slide to obtain the mucosa scraping.

For the histological study, 3-cm sections from the ileum were removed, opened longitudinally, washed thoroughly with sterile PBS and fixed by immersion in a 4% formaldehyde solution (Carlo-Erba Reagents; Sabadell, Spain).

Blood samples were centrifuged (3,000 × g for 15 min at 4°C) after 4 h refrigeration, and the serum obtained was divided into different aliquots and stored at −20°C.

### Analytical procedures

For *Salmonella* bacteria counts (Trial 1), all samples were transferred (1:10) to buffered peptone water. Quantitative assessment was made by seeding serial dilutions of the samples 10^−2^, 10^−4^, and 10^−6^ in Xylose-Lactose-Tergitol-4 plates (Merck; Madrid, Spain). Randomly chosen positive isolates were identified by means of the API20E system (Bio-Mérieux; Barcelona, Spain). With this scheme, animals were given a level as following: negative, for animals with no *Salmonella* growing at 10^2^ dilutions (<10^3^ cfu/g); low, for animals with counts from 10^3^ to 10^4^ cfu/g; medium, for animals with counts from 10^5^ to 10^6^ cfu/g, and high, for counts from 10^7^ to 10^8^ cfu/g.

For enterobacteria and coliform counts (Trial 2), samples were serially diluted in Lactated Ringer's Solution (Sigma-Aldrich; Madrid, Spain) from 10^−4^ up to 10^−8^ and seeded in MacConkey agar (Oxoid; Madrid, Spain) and eosin methylene blue agar (Scharlab; Barcelona, Spain). The plates were incubated for 24 h at 37°C, and colonies were manually counted.

Moreover, in the second trial, *E. coli* K88 was quantified in colonic digesta by real-time PCR (quantitative PCR [qPCR]) using SYBR green dye. DNA from colonic samples (~250 mg) was extracted and purified using the commercial QIAamp DNA stool minikit (Qiagen; West Sussex, United Kingdom). The recommended lysis temperature was increased to 90°C, and a posterior incubation step with lysozyme was added (10 mg × mL^−1^, 37°C, 30 min) in order to improve the bacterial cell rupture. The DNA was eluted in 200 mL of Qiagen buffer AE and stored at −80°C until use. A SYBR green qPCR targeting the gene coding the F4 fimbria of *E. coli* K88 was performed according to the procedure described by Hermes et al. (2013). Results are expressed as cfu/g of fresh matter (FM) and log of F4 gene copies/g FM.

For probiotic detection, DNA was extracted from previously pre-treated colonic samples with a commercial kit v-DNA reagent following the manufacturer's instructions (Geniul; Terrassa, Spain. Doc. Code 450000112). Briefly, samples were re-suspended in 1 mL of v-DNA buffer and centrifuged (13,000 × g for 5 min at 4°C). After that, incubation (90°C, 10 min) with 200 μL of v-DNA reagent was performed in a shaking incubator and finally DNA was suspended in 600 μL of v-DNA buffer. DNA obtained from pure cultures of the probiotic was used for construction of the standard curves. Bacterial cultures were grown overnight in anaerobiosis with MRS broth, and serial 1:10 dilutions were performed in sterile MRS. DNA from dilution −1 (7.5 × 10^7^ cfu/mL) to −6 (7.5 × 10^2^ cfu/mL) was extracted with QIAamp DNA minikit (Qiagen) and used as standard curve. For the qPCR, a strain-specific probe designed for the 16s RNA gene was used ([6FAM]CCGGTTAGTCCTCTACCGTACGCAAGC[TAM]) (Sigma-Aldrich; Madrid, Spain) and an amplicon of 234 bp was obtained by the following primers (Forward: 5′-CGCCGGTGCCAGTCA-3′; Reverse 5′-CACAGCGGGCAGATCGGTAT-3′) (Sigma-Aldrich; Madrid, Spain). The master mix used was 5x Hot Firepol Probe qPCR Mix Plus (no ROX) (Solis BioDyne; Tartu, Estonia) and reaction conditions for amplification of DNA were 95°C for 15 min and 45 cycles of 95°C for 15 s and 60°C for 1 min. Real-time PCR was performed with the ABI 7900 HT Sequence Detection System (PE Biosystems) using optical-grade ninety-six-well plates. The minimum level of detection was established in 3.3 × 10^3^ cfu/g of colonic sample.

The SCFA and lactic acid analyses were performed by gas chromatography, after the samples were submitted to an acid-base treatment followed by an ether extraction and derivatization with *N*-(*tert*butyldimethylsilyl)-*N*-methyl-trifluoroacetamide (MBTSTFA) plus 1% *tert*-butyldimethylchlorosilane (TBDMCS) agent, using the method of Richardson et al. (1989), modified by Jensen et al. (1995).

The concentrations of NH_3_ were determined with the aid of a gas-sensitive electrode (Hatch Co.; Colorado, USA) combined with a digital voltmeter (Crison GLP 22, Crison Instruments, S.A.; Barcelona, Spain). Three grams of acidified content were diluted (1:2) with 0.16M NaOH, after homogenization samples were centrifuged (1,500 × g) for 10 min. The ammonia released was measured in the supernatants as a different voltage in mV according to a procedure previously described in Hermes et al. (2009), which was adapted from Diebold et al. (2004).

Serum concentrations of Tumor Necrosis Factor-α (TNF-α) were determined by Quantikine Porcine TNF-α kits (R&D Systems; Minneapolis, USA) according to the manufacturer's instructions. Pig major acute-phase protein (Pig-MAP) concentration was determined by a sandwich-type ELISA (Pig MAP Kit ELISA, Pig CHAMP Pro Europe S.A.; Segovia, Spain) as described in Saco et al. (2011). In the *Salmonella* trial, serological antibodies of *Salmonella* were tested by ELISA *Salmonella* Herdcheck (Idexx; Hoofddorp, Netherlands) to determine positive animals. Cut-off for positivity was established in optic density ≥40%.

Tissue samples for morphological measures were dehydrated and embedded in paraffin wax, sectioned 4-μm thickness and stained with hematoxylin and eosin. Measurements of 10 different villus-crypt complexes per sample were performed with a light microscope (BHS, Olympus; Barcelona Spain) using the technique described in Nofrarías et al. (2006).

Chemical analyses of the diets, including dry matter (DM), ash, crude protein and diethyl ether extract, were performed according to Association of Official Agricultural Chemists standard procedures (AOAC International, 1995). Neutral-detergent fiber and acid-detergent fiber were determined according to the method of Van Soest et al. (1991).

### Statistical analysis

Results from both trials are expressed as lsmeans with their standard errors unless otherwise stated (microbiological counts were transformed [log] for analysis). A two-way ANOVA was used to examine the effect of the experimental challenge and probiotic treatment, as well as the interaction between the two (only included when significant). The general linear and mixed models of SAS (SAS Institute, 1990; Cary, NC, USA) were used to analyze the effect of experimental treatments as well as Fisher's exact test on microbiological data, to analyze the frequencies of positive animals as contingency tables.

When treatment effects were established, treatment means were separated using the probability-of-differences function adjusted by Tukey–Kramer. The pen was considered the experimental unit for analysis, and random effect was used to account for variation between pens. The α-level used for the determination of significance for all of the analysis was *P* = 0.05. The statistical trend was also considered for *P* < 0.10.

## Results

In general, the trials proceeded as expected. Animals used in both studies showed a good health status at the beginning of the experiment. In the *Salmonella* trial, none of the animals seeded *Salmonella* in feces on arrival, and serological analysis confirmed that animals had not been exposed to *Salmonella* prior to the day of inoculation, all being sero-negative during the whole trial. In the second trial (ETEC K88), one death was registered in the CN group on Day 4 (before the challenge). Death was attributed to post-weaning stress, as the animal was the smallest pig of the pen and had previously shown symptoms of apathy. No antibiotic treatment was administered to any of the animals in the trial.

### Probiotic detection

The ability of the probiotic strain *B. longum* subsp. infantis CECT 7210 to colonize the gut was evaluated by analyzing the probiotic strain by qPCR in the colonic content on Day 8/9 PI. The bacteria was detected in 70% of the treated animals (2.66 × 10^5^ cfu/g for NP and 4.11 × 10^4^ cfu/g for CP in Trial 1; 4.05 × 10^4^ cfu/g for NP and 5.57 × 10^4^ cfu/g for CP in Trial 2) and in none of the non-treated ones (NN and CN). Levels quantified were near the detection limit of the method (established at 3.4 × 10^3^ cfu/g). No significant differences were seen related to the challenge in the number of positive animals or detected concentrations.

### Animal performance

**Figure 1:**
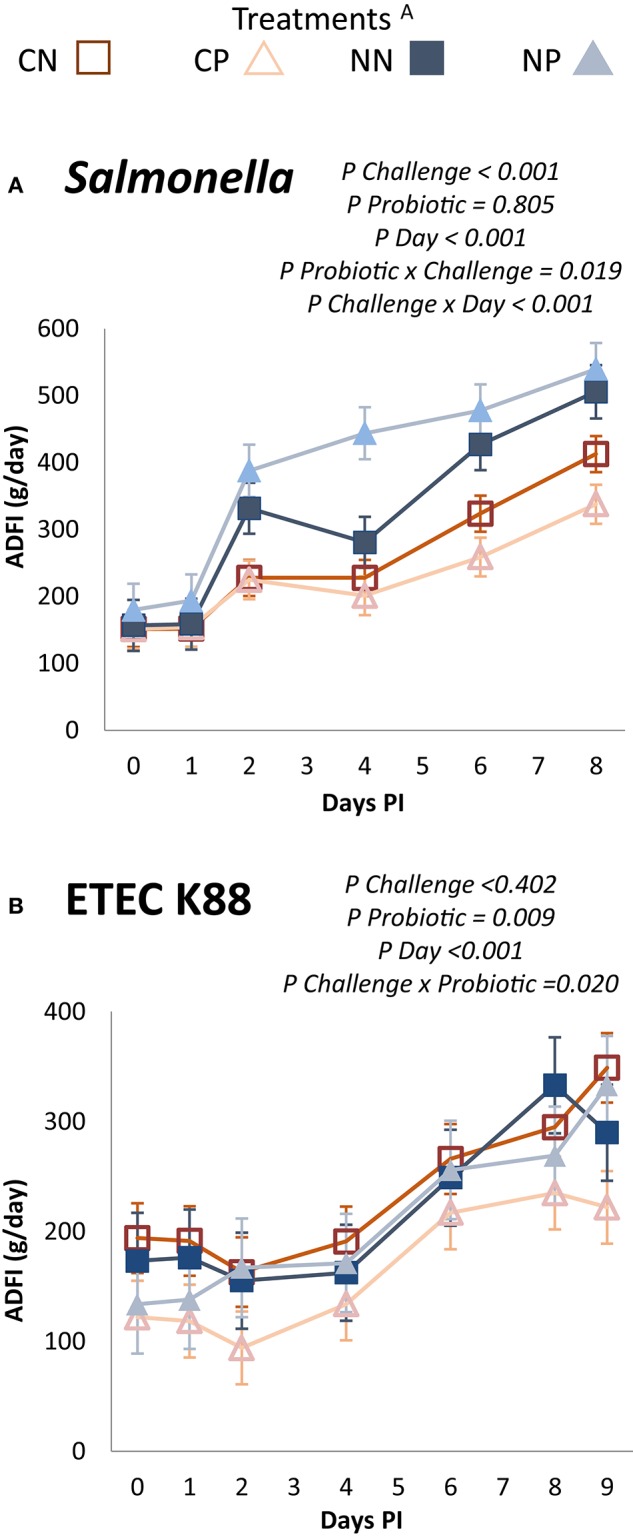
**Average daily feed intake of the post-inoculation period**. Experimental days 8 to 16 (0 to 8 PI) for Trial 1: *Salmonella* **(A)** and 5 to 14 (0 to 9 PI) for Trial 2: ETEC K88 **(B)**. ^A^Treatments: CN, challenged + no probiotic; CP, challenged + probiotic; NN, no challenge + no probiotic; NP, no challenge + probiotic. *n* = 8 for groups CN and CP, *n* = 4 for groups NN and NP. Interactions only included when significant.

Effects of the experimental treatments on BW, ADG, and ADFI are shown in Table 1. The *Salmonella* challenge negatively affected final BW, ADFI, and ADG in the post-challenge period, while the ETEC K88 challenge did not significantly modify any of those parameters. Probiotic treatment did not show significant effects on the studied parameters despite an interaction (*P* = 0.035) observed in the ETEC K88 trial for ADFI during the adaptation period. Before the challenge, the CP group unexpectedly showed a lower ADFI than did its control (CN). Daily registers of ADFI during the post-challenge period are shown in Figure 1. As seen before, the oral challenge with *Salmonella*, but not the ETEC K88 challenge, promoted a significant reduction in the intake. In both trials, a significant interaction challenge x probiotic (*P* = 0.019 for Trial 1 and *P* = 0.020 for Trial 2) was recorded, the probiotic promoting a higher feed intake in the non-challenged animals and a decrease in the challenged ones.

**Table 1:** **Animal performance in ***Salmonella*** and ETEC K88 trials**.

	**Treatments**^**a**^				***P*****-value**			
	**CN**	**CP**	**NN**	**NP**	**RSD^**b**^**	**Challenge**	**Probiotic**	**Interaction**
**TRIAL 1:** ***Salmonella***								
**BW**^c^ **(kg)**								
Initial	7.88	7.88	7.95	7.87	0.131	0.590	0.502	0.516
Final	9.77	9.44	10.91	11.15	0.645	<0.001	0.861	0.318
**ADFI**^d^ **(g/d)**								
Pre-inoculation^e^	179	170	183	183	42.0	0.659	0.812	0.812
Post-inoculation^f^	353	330	458	513	70.0	<0.001	0.598	0.216
**ADG**^g^ **(g/d)**								
Pre-inoculation^e^	94	58	90	103	43.8	0.290	0.539	0.214
Post-inoculation^f^	156	145	288	345	62.0	<0.001	0.399	0.215
**TRIAL 2: ETEC K88**								
**BW**^c^ **(kg)**								
Initial	6.80	6.77	6.72	6.78	0.553	0.883	0.973	0.855
Final	8.74	7.78	8.25	8.35	1.078	0.937	0.184	0.271
**ADFI**^d^ **(g/d)**								
Pre-inoculation^e^	151	84	115	130	42.1	0.787	0.165	0.035
Post-inoculation^f^	283	231	270	278	75.3	0.610	0.510	0.378
**ADG** ^g^ **(g/d)**								
Pre-inoculation^e^	36	–11	23	10	50.5	0.865	0.186	0.434
Post-inoculation^f^	200	119	163	173	76.3	0.808	0.294	0.183

a *Treatments: CN, challenged + no probiotic; CP, challenged + probiotic; NN, no challenge + no probiotic; NP, no challenge + probiotic*.

b *Residual standard deviation*.

c *Body weight*.

d *Average Daily Feed Intake*.

e *Experimental days 0 to 7 for Trial 1 and 0 to 4 for Trial 2*.

f *Experimental days 8 to 16 (0 to 8 PI) for Trial 1 and 5 to 14 (0 to 9 PI) for Trial 2*.

g *Average Daily Gain. n = 8 for groups CN and CP, n = 4 for groups NN and NP*.

### Clinical signs

**Figure 2:**
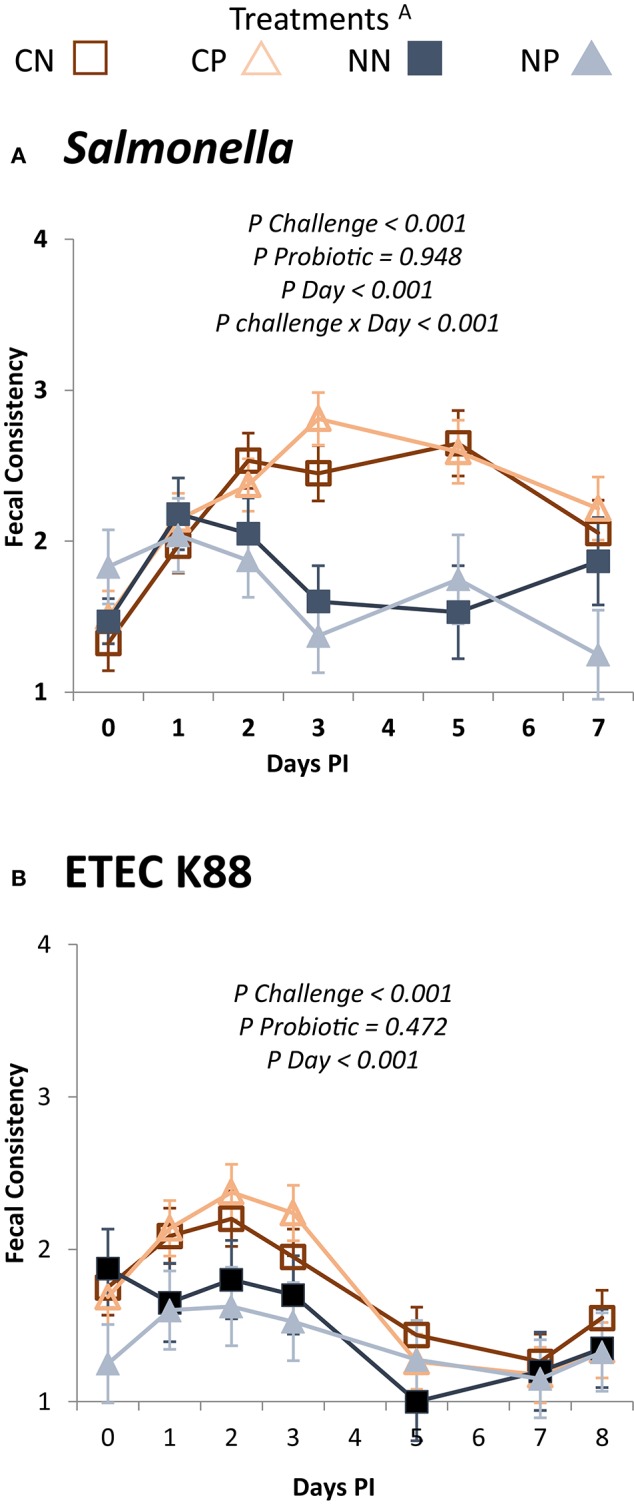
**Evolution of the mean fecal scores in the different experimental groups during the post-challenge period**. Experimental days 8 to 16 (0 to 7 PI) for Trial 1: *Salmonella* **(A)** and 5 to 14 (0 to 8 PI) for Trial 2: ETEC K88 **(B)**. ^A^Treatments: CN, challenged + no probiotic; CP, challenged + probiotic; NN, no challenge + no probiotic; NP, no challenge + probiotic. *n* = 8 for groups CN and CP, *n* = 4 for groups NN and NP. Interactions only included when significant.

Figure 2 shows the evolution of fecal consistency after the challenge in both trials. Pathogen inoculation significantly affected fecal scores, with more liquid feces in both trials (*P* challenge <0.001). However, whereas the *Salmonella* challenge promoted diarrhea with scores between 2 and 3, the ETEC K88 challenge only promoted a slight fecal inconsistency, with scores that were rarely above two. Moreover, there was a clear, differenciated pattern response after the inculation day between challenged and non-challenged animals, as in the *Salmonella* trial the interaction between challenge and day was significant (*P* < 0.001), but the pattern was very similar in the ETEC K88 trial. Regarding the probiotic treatment, no significant differences in fecal consistency were found in any of the trials.

A moderate increment in rectal temperature was seen due to the *Salmonella* challenge (39.3 ± 0.05°C vs. 39.6 ± 0.04°C, *P* = 0.005), while this increment was not significant in the ETEC K88 trial (38.9 ± 0.05°C vs. 39.1 ± 0.06°C, *P* = 0.180). No differences were detected due to probiotic treatment.

### Microbial analysis

**Figure 3:**
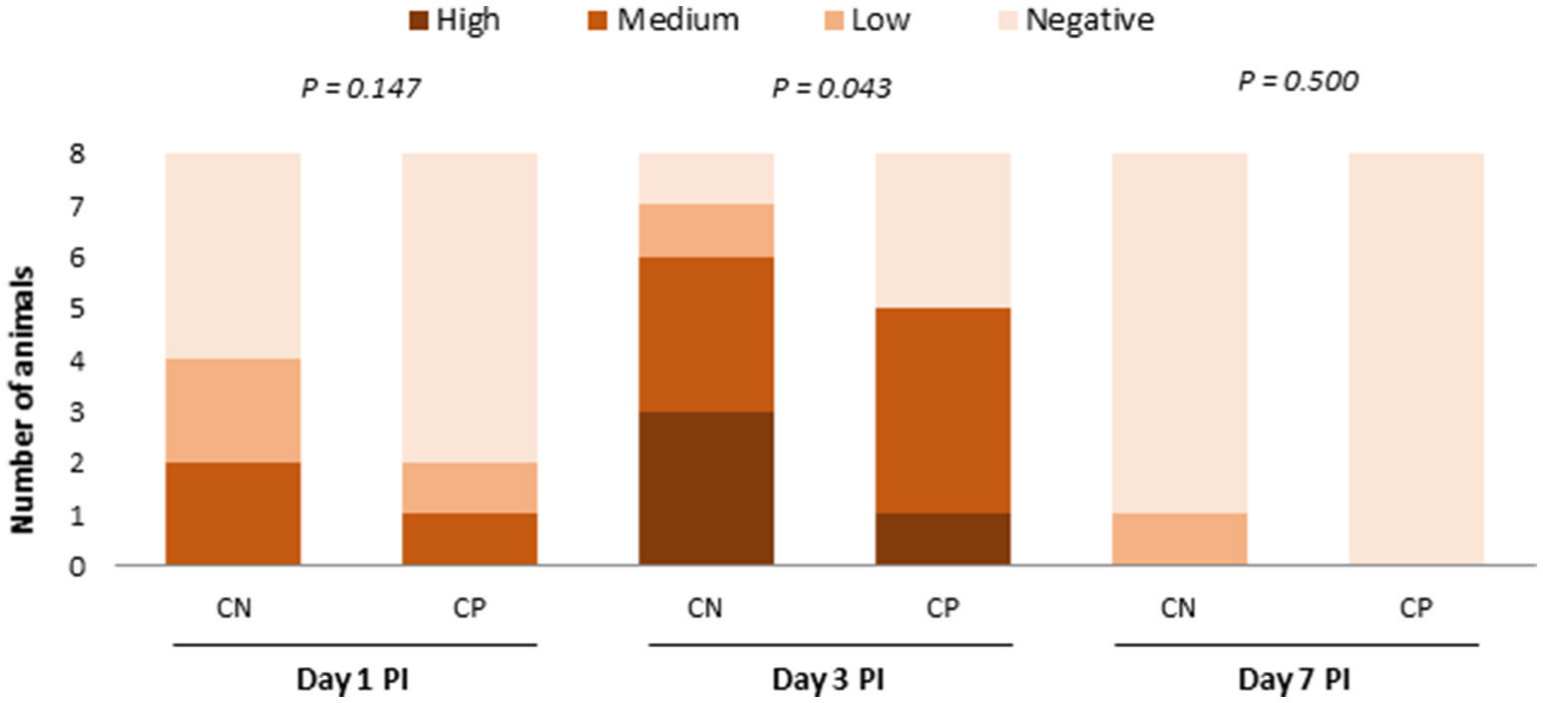
**Number of animals in the different range levels of fecal excretion of ***Salmonella*** at 1, 3, and 7 days post-challenge (Negative 0–10^**2**^ cfu/g, Low 10^**3**^–10^**4**^ cfu/g, Medium 10^**5**^–10^**6**^ cfu/g, and High 10^**7**^–10^**8**^ cfu/g)**. CN, challenged + no probiotic; CP, challenged + probiotic. *n* = 8 for groups CN and CP.

In the first trial, none of the animals seeded *Salmonella* on arrival, and non-challenged piglets remained negative along the whole study. Figure 3 shows the evolution of *Salmonella* counts along the post-challenge period in the challenged group. Challenged animals treated with probiotic had lower fecal excretions of *Salmonella* on Day 3 PI (*P* = 0.043).

**Figure 4:**
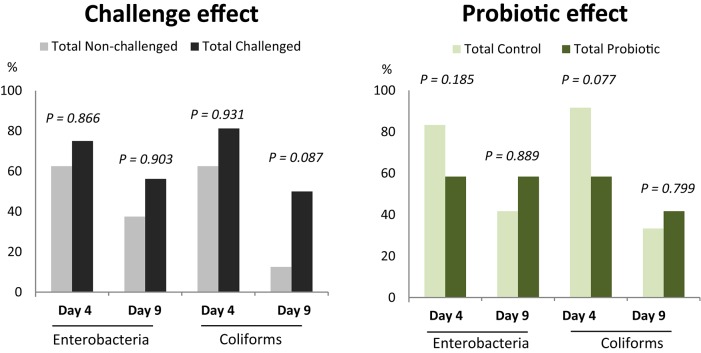
**Percentage of animals in ETEC K88 trial with countable plate counts of enterobacteria or coliforms in ileal scrapings (>10^**5**^ CFU/g)**. Main effects: challenge (*n* = 16 for total challenged animals, *n* = 8 for total non-challenged animals) and probiotic (*n* = 12 for probiotic and control animals).

In the ETEC K88 trial, no significant differences were seen on the arrival day or before the inoculation, in fecal enterobacteria or coliform plate counts (data not shown). Table 2 shows the microbiological analysis on Days 4 and 9 PI in feces and colon digesta. No significant differences related to the experimental treatments were found in enterobacteria or coliform plate counts in feces. Nevertheless, the qPCR aiming to target the inoculated ETEC K88 bacteria in colonic content did show an increase of copy numbers of the F4 fimbria K88 gene in the challenged groups on Day 4 PI (*P* = 0.029). These differences were not maintained on Day 9 PI, when levels decreased in the challenged group near the detection limit of the method (log 3.974 gene copies/g). No differences were found related to probiotic administration. Regarding the microbial plate counts in the ileal scrapings, around 30% of the samples on Day 4 PI and 50% on Day 8 PI were below the minimum level of detection (10^5^ cfu/g). When analyzing the frequencies of animals with countable numbers of enterobacteria or coliforms in ileal scrapings by main effects (challenge effect or probiotic effect, Figure 4), there was a trend for a higher percentage of animals with countable coliforms on Day 9 PI in the challenged group (*P* = 0.087), and the probiotic trended to diminish the percentage of animals with countable coliforms on Day 4 PI (*P* = 0.077). No interaction effects were found.

**Table 2:** **Effects of ETEC K88 trial on the plate counts of total coliforms and enterobacteria in fecal samples (log cfu/g fresh matter [FM]) and F4 gene copy numbers of ***E. coli*** K88 in colon digesta (log of F4 gene copies/g FM)**.

	**Treatments**^**a**^				***P*****-value**			
	**CN**	**CP**	**NN**	**NP**	**RSD^b^**	**Challenge**	**Probiotic**	**Interaction**
**FECES**								
**Enterobacteria (cfu/gFM)**								
Day 4 PI	8.24	8.31	8.48	9.09	1.339	0.390	0.561	0.644
Day 9 PI	7.75	8.31	8.21	8.12	1.114	0.635	0.784	0.511
**Total Coliforms (cfu/gFM)**								
Day 4 PI	8.13	7.83	8.26	8.15	1.009	0.613	0.639	0.824
Day 9 PI	7.5	7.89	8.21	8.08	1.064	0.345	0.787	0.581
**COLON DIGESTA**								
***E. coli*** **K88 (log copies/gFM)**								
Day 4 PI	5.38	6.00	4.25	4.14	1.464	0.029	0.692	0.571
Day 9 PI	4.70	5.11	4.78	4.90	0.556	0.786	0.285	0.556

a *Treatments: CN, challenged + no probiotic; CP, challenged + probiotic; NN, no challenge + no probiotic; NP, no challenge + probiotic*.

b *Residual standard deviation. n = 8 for groups CN and CP, n = 4 for groups NN and NP*.

### Changes in fermentative activity

Table 3 shows the changes promoted by the experimental treatments on the main colonic fermentation products of both trials (ileal data and SCFA molar ratios can be seen in supplementary material [Supplementary Table ST.2 and Supplementary Figure SF.1]).

**Table 3:** **Colonic ammonia concentration and fermentation products for Days 4 and 8 post-inoculation (PI) in ***Salmonella*** and ETEC K88 trials**.

	**Treatments**^**a**^						***P*****-value**		
	**Days PI**	**CN**	**CP**	**NN**	**NP**	**RSD^b^**	**Challenge**	**Probiotic**	**Interaction**
**TRIAL 1:** ***Salmonella***									
NH_3_(mmol/L)	4	12.44	10.46	9.34	7.3	4.051	0.094	0.271	0.987
	8	12.5	12.49	10.19	7.03	5.165	0.097	0.488	0.489
SCFA (mmol/kg)	4	116.5	90.2	123.3	141.3	17.49	<0.001	0.587	0.008
	8	142.3	129.9	131.5	142.1	16.95	0.925	0.905	0.131
Lactic acid (mmol/kg)	4	2.48	4.48	2.45	2.3	6.926	0.717	0.762	0.723
	8	1.18	3.59	0.9	3.58	4.982	0.948	0.251	0.949
**TRIAL 2: ETEC K88**									
NH_3_(mmol/L)	4	14.3	13.5	20.3	14.0	4.67	0.122	0.093	0.197
	9	15.2	27.6	16.7	18.5	11.52	0.457	0.168	0.300
SCFA (mmol/kg)	4	130.7	124.2	113.9	130.4	14.57	0.409	0.435	0.082
	9	147.3	142.0	123.5	136.0	22.00	0.133	0.711	0.363
Lactic acid (mmol/kg)	4	9.05	4.14	7.65	3.02	7.37	0.698	0.151	0.965
	9	2.87	1.65	7.02	2.97	3.75	0.107	0.120	0.392

a *Treatments: CN, challenged + no probiotic; CP, challenged + probiotic; NN, no challenge + no probiotic; NP, no challenge + probiotic*.

b *Residual standard deviation. n = 8 for groups CN and CP, n = 4 for groups NN and NP*.

The *Salmonella* challenge increased ammonia concentration in ileum on Day 4 PI (1.5 vs. 2.3 mmol/l; *P* = 0.033) and showed a trend to increase it in the colon (*P* = 0.094 and *P* = 0.097 for Days 4 PI and 8 PI, respectively). No significant differences related to the experimental treatments were detected in any of the rest of ileal parameters analyzed (pH, SCFA, and lactic acid). In colonic content, a significant decrease in the total concentration of SCFA (*P* < 0.001) was seen with the challenge on Day 4 PI, more pronounced in animals receiving the probiotic treatment (*P* challenge × probiotic = 0.008). This pattern was also found on Day 8 PI with numerical differences (*P* challenge × probiotic = 0.131). Molar ratios of SCFA (See Supplementary Figure SF.1) were not significantly modified by any of the treatments although trends for interactions were seen on Day 4 PI for propionic acid (*P* challenge x probiotic = 0.068) and butyric acid (*P* challenge × probiotic = 0.092). In both cases, the molar proportions in the challenged animals that received the probiotic remained closer to the mean values registered for the non-challenge animals.

In the ETEC K88 trial, pathogen inoculation significantly affected ileal fermentation on Day 9 PI; pH was reduced (6.4 vs. 6.1, *P* < 0.001) while acetic acid and lactic acid (the main fermentation products) tended to be increased (3.4 vs. 9.6 mmol/kg for acetic acid, *P* = 0.051 and 12.7 vs. 37.3 mmol/kg for lactic acid, *P* = 0.076). In colonic digesta, ETEC K88 provoked a numerical increase in pH (*P* = 0.103) on Day 4 PI, with an increase in the molar ratio of propionic (25.8 vs. 29.0%; *P* = 0.015) and a decrease in branched-chain fatty acids (1.69 vs. 1.07%; *P* = 0.042). The probiotic administration significantly increased ileal pH on Day 4 PI (6.32 vs. 6.18, *P* = 0.024) and also trended to increase pH in the colon (5.91 vs. 6.00, *P* = 0.071). Moreover, a trend to reduce ammonia concentration was also seen with the probiotic on Day 4 PI in the colon (*P* = 0.093) and on Day 9 PI in the ileum (1.07 vs. 0.79 mmol/L, *P* = 0.058). Regarding changes in molar proportions with the probiotic, a significant increase of the branched-chain fatty acids molar percentage was seen with the probiotic treatment on Day 9 PI (1.35 vs. 1.99%; *P* = 0.039). Different interactions were registered on Day 4 PI. In the ileum, probiotic trended to decrease lactic acid in challenged animals and to increase it in non-challenged animals (*P* challenge × probiotic = 0.052). In colonic content, the probiotic trended to increase the total SCFA concentration only in the non-challenged animals (*P* challenge × probiotic = 0.082) with increases in the acetic acid proportion in the challenged animals and decreases in the non-challenged ones (*P* challenge × probiotic = 0.017, See Supplementary Figure SF.1). A contrary interaction was seen for molar ratios of propionic and butyric acids (*P* challenge × probiotic = 0.099 and *P* = 0.056, respectively).

### Immune response

Table 4 shows the serological concentrations of the acute-phase proteins Pig-MAP and the pro-inflammatory cytokine TNF-α. In the *Salmonella* trial, both indexes were significantly increased by the challenge on Days 4 (*P* < 0.01) and 8 PI (*P* < 0.03), while in the ETEC K88 trial only TNF-α responded to the challenge on Day 4 PI (*P* = 0.003). The probiotic treatment did not significantly modify any of the parameters, although in the *ETEC K88* trial a significant interaction (*P* = 0.022) was found in Pig-Map values on Day 4 PI, when the probiotic treatment increased this index in the challenged animals but decreased it in non-challenged ones.

**Table 4:** **Effects on serum levels of pro-inflammatory cytokine TNF-α and acute-phase protein Pig-MAP on Days 4 and 8 post-inoculation in ***Salmonella*** and ETEC K88 trials**.

	**Treatments**^**a**^						***P*****-value**		
	**Days PI**	**CN**	**CP**	**NN**	**NP**	**RSD^b^**	**Challenge**	**Probiotic**	**Interaction**
**TRIAL 1:** ***Salmonella***									
Pig-Map (mg/ml)	4	1.65	2.57	0.87	0.74	0.907	0.003	0.326	0.194
	8	1.33	1.68	0.80	0.64	0.762	0.027	0.788	0.449
TNF-α (pg/ml)	4	146.2	155.0	95.8	84.5	37.01	0.001	0.938	0.542
	8	128.1	134.1	87.2	82.7	27.95	0.001	0.950	0.673
**TRIAL 2: ETEC K88**									
Pig-Map (mg/ml)	4	0.80 ^a^^,^^b^	0.92 ^a^	0.87 ^a^^,^^b^	0.67 ^b^	0.147	0.163	0.551	0.022
	9	0.84	1.14	0.77	0.75	0.599	0.379	0.603	0.539
TNF-α (pg/ml)	4	83.9	79.2	64.0	49.9	16.85	0.003	0.218	0.536
	9	92.3	92.2	90.1	77.2	17.97	0.284	0.516	0.427

a *Treatments: CN, challenged + no probiotic; CP, challenged + probiotic; NN, no challenge + no probiotic; NP, no challenge + probiotic*.

b *Residual standard deviation. n = 8 for groups CN and CP, n = 4 for groups NN and NP*.

### Intestinal morphology

The histomorphological results of the ileum on Day 4 PI are summarized in Table 5. Results for Day 8/9 PI are not shown in the table. The *Salmonella* challenge caused a decrease in villus height (*P* < 0.001), deeper crypt depth (*P* = 0.007), and worse villus:crypt ratio (*P* < 0.001) on Day 4 PI. A significant interaction was seen on Day 8 PI in crypt depth that was increased by the probiotic in the non-challenged animals and decreased in the challenged ones (212, 240, 229, and 203 μm NN, NP, CN, and CP, respectively; *P* = 0.017). Moreover, other trends for similar interactions were seen in the villus:crypt ratio on Day 4 PI (*P* = 0.107) and Day 8 PI (*P* = 0.091). The *Salmonella* challenge increased intra-epithelial lymphocytes (IEL) both days PI (*P* = 0.042 for Day 4 PI and 1.71 vs. 2.18, *P* = 0.046 for Day 8 PI) and mitosis in crypts on Day 4 PI (0.29 vs. 0.43 cel/100 μm, *P* = 0.035). Probiotic also had significant effects on Day 8 PI when it increased IEL (1.80 vs. 2.09 cel/100 μm, *P* = 0.002) and decreased goblet cells (GC) (1.31 vs. 1.05 cel/100 μm, *P* = 0.025).

**Table 5:** **Histological determinations in ileum on Day 4 PI in ***Salmonella*** and ETEC K88 trials**.

	**Treatments**^**a**^					***P*****-value**		
**Day 4 PI**	**CN**	**CP**	**NN**	**NP**	**RSD^b^**	**Challenge**	**Probiotic**	**Interaction**
**TRIAL 1:** ***Salmonella***								
Villus height (μm)	186	141	250	296	46.9	<0.001	0.992	0.361
Crypt depth (μm)	244	245	206	218	24.5	0.007	0.562	0.581
Ratio Villus:Crypt	0.78	0.58	1.22	1.37	0.242	<0.001	0.818	0.107
IEL^c^ (N° cel/100 μm)	2.09	2.03	0.75	1.44	1.027	0.042	0.482	0.407
**TRIAL 2: ETEC K88**								
Villus height (μm)	224	223	252	260	49.7	0.152	0.886	0.818
Crypt depth (μm)	205	213	187	207	20.7	0.194	0.144	0.504
Ratio Villus:Crypt	1.11	1.05	1.34	1.25	0.258	0.066	0.524	0.877
IEL^c^ (N° cel/100 μm)	2.64	2.74	1.79	2.36	0.420	0.004	0.091	0.224

a *Treatments: CN, challenged + no probiotic; CP, challenged + probiotic; NN, no challenge + no probiotic; NP, no challenge + probiotic*.

b *Residual standard deviation. n = 8 for groups CN and CP, n = 4 for groups NN and NP*.

c *IEL = Villus intraepithelial lymphocytes*.

In the ETEC K88 trial, the challenge tended to decrease the villus:crypt ratio on Day 4 PI (*P* = 0.066), increased IEL both days (*P* = 0.004 Day 4 PI and 2.08 vs. 2.49, *P* = 0.104 Day 9 PI) as well as GC on Day 9 PI (0.91 vs. 1.22 cel/ 100, *P* = 0.093). The probiotic treatment trended to increase IEL on Day 4 PI (*P* = 0.091), and an interaction was seen on Day 9 PI in villus height (*P* = 0.017), and villus:crypt ratio (*P* = 0.006) with lower values in CP and increased ones in NP. No differences related to the experimental treatments were detected in mitosis.

## Discussion

The aim of this study is to determine if the administration of the probiotic strain *B. longum* subsp. *infantis* CECT 7210 is able to enhance health at early life stages and, moreover, if it confers protection against common opportunistic digestive pathogens such as *Salmonella* Typhimurium or ETEC K88. To assess this objective, the probiotic was tested in two different trials, one for each pathogen, and a different clinical outcome was obtained for each challenge. In the *Salmonella* trial, nearly all parameters evaluated responded significantly to the challenge, with the animals presenting clear clinical signs of acute self-limiting diarrhea whereas, in the ETEC K88 challenge, effects were milder and no severe cases were seen, the evaluated parameters being weakly affected. Hence, at the end, we were able to test the probiotic in a wide range of intestinal disease.

In relation to the ability of bifidobacteria to inhibit pathogens, Shu et al. (2000, 2001) observed, in a piglet model, that *Bifidobacterium lactis* conferred protection against *E. coli* and *Salmonella* (among other intestinal pathogens). However, it did not completely reduce the pathogenic colonization. In addition, Knol et al. (2007) associated an increase of bifidobacteria with reductions of the presence of clinically relevant pathogens in formula-fed pre-term infants. These protective effects against pathogens have mainly been attributed to a pluripotent stimulatory effect on the immune system (Gill et al., 2001; Medina et al., 2007; Takahashi et al., 2006), although some other modes of action could explain their antimicrobial activity like the production of organic acids (Saulnier et al., 2009), bacteriocines, and bacteriocine-like substances (Cheikhyoussef et al., 2008), and the capacity to inhibit the pathogenic adhesion to enterocytes or prevent bacterial translocation (Gagnon et al., 2004; Searle et al., 2009).

In the *in vivo* study herein presented, the administration of a single daily dose (10^9^ cfu) of *B. longum* subsp. infantis CECT 7210 was not able to fully prevent pathogen colonization. Nevertheless, it was able to reduce pathogen loads, particularly the load of *Salmonella* in the fecal content and the number of animals with high numbers of coliforms attached to their ileal mucosa.

The probiotic also showed some particularly beneficial effects on the animals. A clear effect on intestinal immune function was seen with an increase in ileal IEL in both challenges. It is well-established that IEL have a relevant role in the gastrointestinal immune system, playing an important role in the regulation of the immune response (Ogra et al., 1994). In particular, other immune effects have been described with the same strain. (Moreno Muñoz et al., 2011) reported an increment of the IgA antibody levels in feces by the inclusion of the probiotic in a murine model and also increases in anti-inflammatory IL-10 have been recorded (unpublished data). In addition, recent works have reported the production of peptides with protease activity by this strain, able to hydrolyze β-casein and produce functional peptides with antirotaviral activity (Chenoll et al., 2015, 2016).

Effects of the probiotic treatment on other parameters evaluated in this study were variable, depending on whether animals were challenged or not, and should be analyzed separately.

In general terms, the effects of the probiotic in weight gains and feed intakes were scarce. Nevertheless, in both trials, during the first week post-challenge, an interaction could be seen in the ADFI, with enhanced feed consumption in the non-challenged animals receiving probiotic and diminution in the challenged ones. Moreover, at the end of the ETEC K88 trial, the challenged animals receiving the probiotic showed almost 1 kg of BW less than their counterparts, despite this difference not being significant. This apparent worse performance of the challenged animals with the probiotic could be explained in the ETEC K88 trial by a random, worse adaptation of the CP group to weaning, as this group unexpectedly decreased their intakes during the first week before the challenge. Nonetheless, this explanation cannot be given for the *Salmonella* trial, which suggests a differential effect of the probiotic in challenged or non-challenged animals.

Regarding colonic fermentation, it could be observed in both trials, on Day 4 PI, that animals treated with the probiotic showed an increase in the SCFA concentrations if they were not challenged, but if they were orally inoculated with the pathogen, SCFA concentration was reduced. In addition, a favorable fermentative environment was detected in non-challenged animals receiving probiotic, with a tendency to increase butyric acid but not in the challenged ones. Although *Bifidobacterium* spp. are not butyrate producing bacteria, it has been proposed that cross-feeding of lactate or acetate produced by bifidobacteria can stimulate the formation of butyrate by other bacteria within the gut community (Belenguer et al., 2006; Van der Meulen et al., 2006; De Vuyst and Leroy, 2011). For instance, Falony et al. (2006) co-cultured fermentations of *B. longum* and two acetate-converting, butyrate-producing colonic bacteria with oligofructose as the sole energy source and observed interspecies interactions. Due to the high complexity of the colon ecosystem, we could not evaluate this metabolic process in our *in vivo* assay. Nevertheless, the higher presence of butyrate reported for the NP group in both trials and the diminution of acetic acid in the same animals registered in the ETEC K88 trial would suggest a possible cross-feeding phenomenon present only in the non-challenged animals. In the challenged animals, gut dysbiosis caused by the challenge had probably disturbed all of these microbial interactions so much that it precluded the probiotic to promote butyrogenic effects. Moreover, histomorphological findings reinforce this theory as probably a higher butyrate presence in the NP group, being the preferred energy source for the colonocyte and a potent differentiating agent (Scheppach, 1994), contributed to the observed increase of the villus:crypt ratio of the non-challenged animals treated with the probiotic.

Furthermore, although statistical significance was only achieved in the ETEC K88 trial, Pig-MAP also responded differentially, with increases in the challenged animals receiving probiotic and decreases in the non-challenged ones on Day 4 PI. In swine, Pig-MAP is a major acute-phase protein, and higher serum concentrations have been related to acute inflammatory processes and also to the extent of tissue injury, expressing strong and protective responses to bacterial infections (Piñeiro et al., 2009). As mentioned above, our strain has been reported to have anti-inflammatory effects (Moreno Muñoz et al., 2011), but no evidence of this effect was seen in the CP group.

All of these results suggest that our probiotic interacted differently in each situation. A better comprehension of these interactions (such as gut health conditions, bacterial populations, and their connections) could bring to light improved application protocols to get over the inconsistent results reported nowadays in scientific literature.

This possible differential behavior of a probiotic under a disease condition is particularly relevant if we wish to use the probiotic therapy to treat intestinal disease. It is not the first case in scientific literature in which uncertainty regarding probiotic use is reported, as it has been recorded that probiotics can be useful in a healthy situation, but detrimental when the intestinal barrier is affected, especially if the patient is in an immuno-compromised situation; which could lead to bacterial translocation and sepsis (Liong, 2008). In humans, these bacterial sepsis for probiotic treatments have been mainly reported for *Lactobacillus* spp. and *Bacillus* spp., as reviewed by Boyle et al. (2006) and Liong (2008) but recently bifidobacterial sepsis have been recorded in preterm infants treated with probiotic (Jenke et al., 2012; Bertelli et al., 2015; Zbinden et al., 2015) and even in an elder man undergoing chemotherapy treatment (Weber and Reynaud, 2015). Trevisi et al. (2008) tried different concentrations of a *Bifidobacterium animalis* in weaning piglets with and without a FOS-based prebiotic and found a positive correlation of the *B. animalis* administration and translocation in mesenteric lymph nodes when FOS was supplemented. The authors speculated about the possibility of an increase in other bacteria promoted by FOS that could also be translocated and thus, contribute to the reported results.

Regarding the safety of the probiotic strain tested in this study, (Moreno Muñoz et al., 2011) tested a 10^9^ cfu dose in an acute ingestion study with immunosuppressed mice, and no bacterial load of *Bifidobacterium* spp. was found in blood, liver, spleen, or mesenteric lymph nodes. Bifidobacteria are generally considered safe and a vast body of literature appeals to protection exercised by these probiotics in avoiding pathogen translocation. Nevertheless, we speculate that in our challenged animals, gut dysbiosis caused by the bacterial challenge, in addition to the weaning syndrome, might have led the probiotic to have a detrimental effect despite the observed reductions in pathogen loads. More research should be addressed to study any possible effect of this probiotic on the barrier integrity and bacterial translocation.

Finally, although the pig has been described as an excellent model for humans (Meurens et al., 2012; Wang and Donovan, 2015), certain limitations of the model should be taken into account when interpreting results. Firstly, the host-specific nature of *B. longum* subsp. *Infantis*. This bacteria is one of the first and most predominant gut colonizers in infants (Underwood et al., 2015) but not in pigs. Moreover, it has co-evolved with humans (Arboleya et al., 2016) and this would explain why *B. infantis* grow better in the presence of human-milk oligosaccharides than in lactose. When human-milk oligosaccharides are present, *B. infantis* increases their adhesion rate to intestinal cells and expression of selected cytokines (Chichlowski et al., 2012). Secondly, gastrointestinal disorders are multifactorial, and therefore it is difficult to fully emulate them in controlled experimental conditions (Rossi et al., 2012). A moderate-high controlled dose of a single intestinal pathogen (even in multiple doses as in our experiment) achieved significant challenge effects (diarrhea, inflammation, histomorphological affections…) and allowed us to test the probiotic in different controlled ranges of intestinal affections. However, this way to be exposed to the pathogen is quite different from what a natural process would be, where a low and continuous exposure is normally expected. Lastly, the way the probiotic is administered can also determine differences in the response. In this study, the probiotic was given in a single bolus every morning at the peak of eating activity, aiming to ensure that piglets received the stated concentration of viable bacteria and that the stomach was full to favor probiotic viability. Nevertheless, the effects could have been different if we had given the probiotic in a continuous pattern, as it would be if added to a milk formula. Considering that the pig is not the natural host for these bacteria, the probiotic probably had few opportunities to persist in the gastrointestinal tract. This could be the explanation for the low numbers of probiotic cells found in the colon, considering that the animals were sampled more than 24 h after the last dose.

To sum up, various consistent effects of the probiotic in both experiments were detected although a different response pattern was seen between challenged and non-challenged animals. The potential of the probiotic was demonstrated by clearly producing a beneficial response in non-challenged animals and by improving their post-weaning situation. Nevertheless, the response of challenged animals treated with probiotic was not always positive although the probiotic consistently reduced the pathogen loads. More in-depth investigation should be performed to better assess the mechanisms for the different response patterns observed and improve probiotic therapeutic protocols. Limitations in the model must be considered when evaluating experiment results and extrapolating the potential of the probiotic to children.

## Conclusions

The probiotic *B. longum* subsp. *infantis* CECT 7210 had a positive effect against pathogens by reducing the fecal excretion of *Salmonella* Typhimurium and the mucosal colonization of coliforms in the ETEC K88 trial. In addition, it produced a stimulation of the intestinal immune system by increasing IEL. Different responses to the probiotic in challenged and non-challenged animals were also recorded, mainly in feed intake, SCFA concentrations, and the villus:crypt ratio that would suggest a distinct effect of the probiotic intervention, depending on the structure of the microbiota and on the integrity of the intestinal barrier. More research is needed for fully guarantee the safety and efficacy of this strain for its use in children with gastrointestinal disorders.

## Author contributions

EB participated in the experimental design, was responsible for the animal trial, laboratory analysis, data analysis, and writing. LC participated in the experimental design, animal trials, data analysis and writing. PL participated in animal trials, data analysis, and writing. MR and JM participated in the experimental design, contributed to data analysis, and writing. SM participated in the experimental design, animal trials, laboratory analysis, data analysis, and writing.

## Funding

This study was supported by CDTI (Spanish Center for the Development of Industrial Technology).

### Conflict of interest statement

This work was partially funded by Laboratorios Ordesa. MR and JM are employees of Laboratorios Ordesa S.L. The others authors declare that the research was conducted in the absence of any commercial or financial relationships that could be construed as a potential conflict of interest

## Footnotes

^1^Directive 2010/63/EU of the European Parliament and of the Council of 22 September 2010 on the protection of animals used for scientific purposes Official Journal.
