# Supplementary material for: Evaluation of the Probiotic Strain Bifidobacterium longum subsp. Infantis CECT 7210 Capacities to Improve Health Status and Fight Digestive Pathogens in a Piglet Model
Source: Front Microbiol. 2017 Apr 11;8:533. doi: 10.3389/fmicb.2017.00533 (PMC5386966; doi:10.3389/fmicb.2017.00533)
Supplement: Supplementary file 1 [file Table1.pdf]

Supplementary Material

Evaluation of the probiotic strain *Bifidobacterium longum* subsp. *infantis* CECT 7210 capacities to improve health status and fight digestive pathogens in a piglet model

Emili Barba-Vidal[1], Lorena Castillejos[1], Paola López-Colom[1], Montserrat Rivero Urgell[2], José A. Moreno Muñoz [2] and Susana M. Martín-Orúe[1*]

***Correspondence:** Susana M. Martín-Orúe. Susana.Martin@uab.cat

Supplementary Table ST.1. Ingredient and nutrient composition of the experimental diets as-fed basis, g/kg.

**Ingredients**	
Maize	280.8
Wheat	170.0
Barley 2 row	150.0
Extruded soybean	122.4
Sweet whey-powder (cattle)	100.0
Fishmeal	50.0
Soybean meal 44	50.0
Whey-powder 50% fat	30.3
Mono-calcium phosphate	21.3
Calcium carbonate (CaCO3)	8.2
L-Lysine HCL	4.5
Vitamin-Mineral Premix [A]	4.0
Sodium chloride (marine salt)	3.0
DL-Methionine 99	2.4
L-Threonine	2.3
L-Triptophane	0.9
**Analyzed composition**	
DM	911.0
CP	174.9
CF	59.4
NDF	92.7
ADF	34.8
Ash	72.5

[A] Provided per kilogram of complete diet: 10,200 IU vitamin A, 2,100 IU vitamin D_3. 39.9 mg vitamin E, 3 mg vitamin K_3, 2 mg vitamin B_1, 2.3 mg vitamin B_2, 3 mg vitamin B_6, 0.025 mg vitamin B_{12}, 20 mg calcium panthotenate, 60 mg nicotinic acid, 0.1 mg biotin, 0.5 mg folic acid, 150 mg Fe, 156 mg Cu, 0.5 mg Co, 120 mg Zn, 49.8 mg Mn, 2 mg I, 0.3 mg Se.
