# Supplementary material for: Evaluation of the Probiotic Strain Bifidobacterium longum subsp. Infantis CECT 7210 Capacities to Improve Health Status and Fight Digestive Pathogens in a Piglet Model
Source: Front Microbiol. 2017 Apr 11;8:533. doi: 10.3389/fmicb.2017.00533 (PMC5386966; doi:10.3389/fmicb.2017.00533)
Supplement: Supplementary file 2 [file Table2.pdf]

Supplementary Material

Evaluation of the probiotic strain *Bifidobacterium longum* subsp. *infantis* CECT 7210 capacities to improve health status and fight digestive pathogens in a piglet model

Emili Barba-Vidal[1], Lorena Castillejos[1], Paola López-Colom[1], Montserrat Rivero Urgell[2], José A. Moreno Muñoz [2] and Susana M. Martín-Orúe[1*]

***Correspondence:** Susana M. Martín-Orúe. Susana.Martin@uab.cat

Supplementary Table ST.2. Ileal pH values, ammonia concentration and fermentation products for Days 4 and 8 post-inoculation (PI) in *Salmonella* and ETEC K88 trials.

		**Treatments** [A]					***P-value***		
	**Days PI**	**CN**	**CP**	**NN**	**NP**	**RSD** [B]	**Challenge**	**Probiotic**	**Interaction**
**Trial 1. *Salmonella***									
**pH**	4	6.14	6.16	6.22	6.32	*0.274*	*0.318*	*0.618*	*0.724*
	8	5.88	5.90	5.84	6.28	*0.281*	*0.175*	*0.070*	*0.096*
**NH_3**	4	2.42	2.34	1.65	1.32	*0.901*	*0.033*	*0.604*	*0.758*
(mmol/L)	8	1.20	0.97	0.63	0.83	*0.601*	*0.183*	*0.958*	*0.421*
**SCFA**	4	5.96	3.83	5.30	4.90	*2.424*	*0.906*	*0.460*	*0.610*
(mmol/kg)	8	3.43	4.01	2.95	4.05	*1.117*	*0.662*	*0.112*	*0.612*
**Lactic acid**	4	32.6	41.3	43.6	15.2	*33.60*	*0.609*	*0.507*	*0.217*
(mmol/kg)	8	71.6	66.0	72.7	33.9	*39.22*	*0.373*	*0.206*	*0.339*
**Trial 2. ETEC K88**									
**pH**	4	6.19	6.28	6.18	6.34	*0.122*	*0.718*	*0.024*	*0.508*
	9	6.24	6.13	6.43	6.39	*0.124*	*<0.001*	*0.181*	*0.500*
**NH_3**	4	3.80	3.72	4.02	3.32	*1.270*	*0.867*	*0.491*	*0.572*
(mmol/L)	9	0.98	0.84	1.15	0.74	*0.321*	*0.777*	*0.058*	*0.335*
**SCFA**	4	5.16	4.68	6.43	5.00	*2.458*	*0.486*	*0.403*	*0.678*
(mmol/kg)	9	10.67	8.52	4.00	2.87	*5.765*	*0.051*	*0.578*	*0.863*
**Lactic acid**	4	63.5	30.9	18.1	40.9	*31.00*	*0.202*	*0.719*	*0.052*
(mmol/kg)	9	33.6	41.1	19.5	5.9	*30.40*	*0.076*	*0.820*	*0.433*

[A] Treatments: CN, challenged + no probiotic; CP, challenged + probiotic; NN, no challenge + no probiotic; NP, no challenge + probiotic. [B] Residual standard deviation. n = 8 for groups CN and CP, n = 4 for groups NN and NP.
