# Supplementary material for: Evaluation of the Probiotic Strain Bifidobacterium longum subsp. Infantis CECT 7210 Capacities to Improve Health Status and Fight Digestive Pathogens in a Piglet Model
Source: Front Microbiol. 2017 Apr 11;8:533. doi: 10.3389/fmicb.2017.00533 (PMC5386966; doi:10.3389/fmicb.2017.00533)
Supplement: Supplementary file 3 [file Image1.PDF]

Supplementary Material

Potential of the probiotic strain *Bifidobacterium longum* subsp. *infantis* CECT 7210 to improve health status and fight digestive pathogens

Emili Barba-Vidal[1], Lorena Castillejos[1], Paola López-Colom[1], Montserrat Rivero Urgell[2], José A. Moreno Muñoz [2] and Susana M. Martín-Orúe[1*]

***Correspondence:** Susana M. Martín-Orúe. Susana.Martin@uab.cat

Supplementary Figure SF.1. Colonic short-chain fatty acids molar ratios.

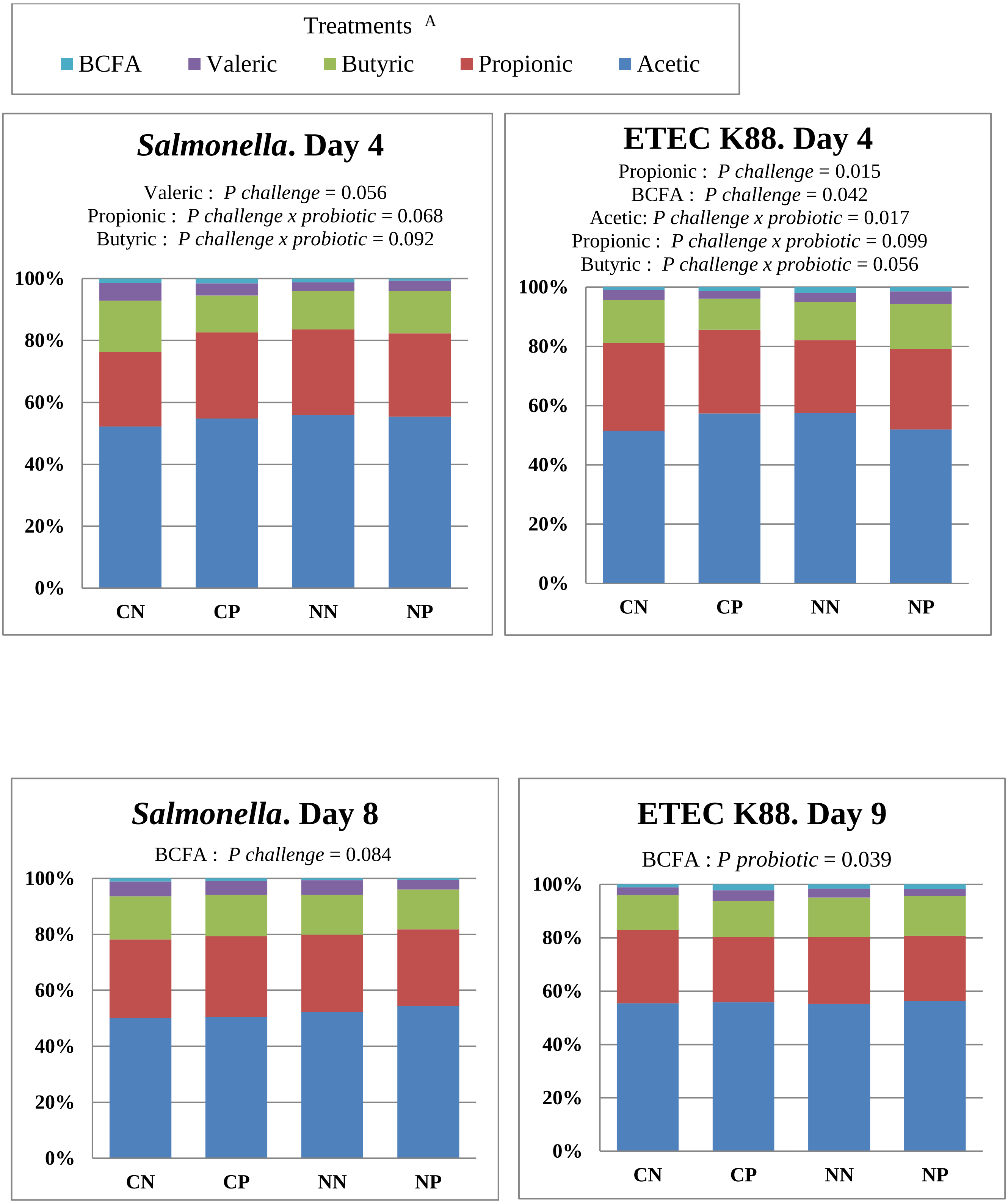

[A] Treatments: CN, challenged + no probiotic; CP, challenged + probiotic; NN, no challenge + no probiotic; NP, no challenge + probiotic.
